# Food Allergy and Asthma: Is There a Link?

**DOI:** 10.1007/s40521-018-0185-1

**Published:** 2018-10-01

**Authors:** Joyce A. M. Emons, Roy Gerth van Wijk

**Affiliations:** 1 Erasmus MC, Department of Pediatrics, Division of Respiratory Medicine and Allergology, University Medical Center Rotterdam, Rotterdam, The Netherlands; 2 Erasmus Medical Center, Department of Internal Medicine, Section of Allergology, University Medical Center Rotterdam, Rotterdam, The Netherlands

**Keywords:** Food hypersensitivity, Anaphylaxis, Asthma, Atopic dermatitis, Prevalence, Comorbidity, Occupational asthma

## Abstract

**Purpose of review:**

To describe and understand the links and interactions between food allergy and asthma

**Recent findings:**

Food allergy and asthma are characterized by an increasing prevalence. Moreover, food allergy and asthma often coexist. Both conditions are associated with each other in different ways. It has been shown that food allergy is a risk factor of developing asthma. Atopic dermatitis appears to be the common denominator in this interaction. Loss-of-function variants of the filaggrin mutation result in an impaired epidermal barrier function and have been shown to be a risk factor for the development of atopic dermatitis, allergies, and asthma. Early introduction of food allergens and optimal treatment of the skin barrier are preventive interventions for the development of food allergy and asthma. Asthma is also a risk factor for the development of severe or even fatal anaphylaxis in patients with food allergy. Isolated asthma is not a feature of a food allergic reaction; however, respiratory symptoms may be part of anaphylactic reactions. In addition, during an allergic reaction to food, non-specific bronchial hyperreactivity may increase. Cross-reactive allergens may be responsible for asthma-associated food allergy. This is particularly true for severe asthma upon ingestion of snail in patients allergic to house-dust mites. Finally, airborne allergens from occupational sources such as wheat, fish, and seafood may induce asthmatic reactions. This phenomenon is sometimes seen in non-occupational settings.

**Summary:**

Food allergy and asthma are interconnected with each other beyond the presence of simple comorbidity. Food allergy precedes and predisposes to asthma, and mutual interactions range from respiratory symptoms and bronchial hyperreactivity during food-induced anaphylaxis to severe asthma due to cross-reactive food allergens and to occupational asthma upon exposure to airborne allergens. Moreover, coexisting asthma in food allergies may result in severe and sometimes fatal anaphylactic reactions.

## Introduction

Asthma is a common global health problem affecting all age groups. Epidemiologic studies have shown that up to 20% of children aged 6–7 year experience a wheezing episode within a year and in adults, global prevalence rates are reported up to 21% [1, 2]. Food allergy prevalence varies widely and this is most likely caused by different methods in how to define a food allergy but also in geographical differences. Prevalence numbers vary from 1 to 2% up to 11% with patient self-reported food allergy even higher up to 35% [3–5]. The most common food allergens in young children are milk (2.5%), egg (1.3%), peanut (0.8%), wheat (0.4%), soy (0.4%), tree nuts (0.2%), fish (0.1%), and shellfish (0.1%) of which most outgrow milk, egg, wheat, and soy by the age of 5 years. The most common food allergies in adults are shellfish (2%), scaled fish (0.4%), peanut (0.6%), and tree nuts (0.5%) which usually persist throughout life [6].

Asthma and food allergy are frequently coexisting and both are increasing in prevalence. In a Dutch cross-sectional study with asthmatic children, half of the parents reported an allergic reaction to any type of food in their child’s history [7]. Several studies showed that asthmatic children have more sensitizations to food allergens compared to the general population and sensitization is associated with increased asthma severity [6, 8, 9]. Schroeder et al. showed that children with a food allergy are more often diagnosed with asthma and there was a stronger association among children with multiple or severe food allergies [10]. A large German retrospective cohort study showed that food allergy was an important risk factor for developing asthma, with an odds ratio of 2.16 [11]. Roberts et al. even reported a higher rate; they showed that children with food allergies are around six times more likely to suffer from severe asthma later in life than children who did not have food allergies [12]. More specifically, it was shown that an egg allergy early in life was associated with a fivefold increased risk for a respiratory allergic disease in childhood [13]. These observations are in line with the atopic march hypothesis in which it is described that in atopic patients, eczema is the first manifestation, followed by food allergy, asthma, and allergic rhinitis [14]. Interestingly, an English study showed that wheezing before the age of 2 years was not significantly related to adult asthma, but a positive skin prick test to egg or milk in the first year of life was a strong risk factor for the development of adult asthma [15].

Food allergy can be classified into IgE and non-IgE-mediated food allergies with the latest being less well described and immunologically less well understood. However, also a non-IgE-mediated food allergy is associated with asthma. In an English study, approximately one third of children with a non-IgE-mediated food allergy (mostly gastrointestinal symptoms) had concomitant asthma [16].

## Risk factors for the development of food allergy and asthma: atopic dermatitis

As mentioned above, food allergy and asthma are closely related and frequently food allergy in early childhood precedes asthma at a later time point in child- or adulthood as described in the atopic march hypothesis [14].

### Atopic dermatitis and the development of food allergy

Another important risk factor for the development of both food allergy and asthma is atopic dermatitis. Prevalence numbers vary in literature, but percentages of 81% are described of pediatric patients with atopic dermatitis that will develop a food allergy later in life. In addition, a systematic review of Tsakok et al. reported an increased risk of six times of food sensitization in patients with atopic dermatitis compared to healthy controls in population-based studies [17••]. They also showed that up to 53% of patients with atopic dermatitis were food sensitized and up to 15% had a challenge proven allergy [17••]. A recent study, the EAT study, which analyzed prevention of food allergy by early introduction of food showed that at 3 months of age, children with atopic dermatitis showed 8.5 times more sensitization to food with a skin prick test compared to babies without atopic dermatitis. This was especially the case for egg and peanut and with increasing severity of atopic dermatitis [18]. Guillet and Guillet studied children with atopic dermatitis and found an increase in prevalence of food allergy with increased severity of eczema [19]. In children with severe atopic dermatitis, 100% sensitization to food was found with a clinical relevance of increase of their atopic dermatitis for almost all patients after intake of food allergens [19].

Based on these and a number of other epidemiologic observations, the dual-allergen-exposure hypothesis was postulated in 2008, which described that oral antigen exposure tends to induce tolerance, whereas skin exposure tends to induce allergic sensitization, leading to food allergy [20]. In line with this early introduction of food allergens in the diet is now advised in young children with atopic dermatitis in combination with optimal treatment and control of the skin barrier.

### Atopic dermatitis and the development of asthma

Atopic dermatitis is found to be a risk factor for asthma as well but studies are sometimes controversial in this. A birth cohort study in the UK showed that participants with atopic dermatitis early in life had a 2–3-fold increased risk of asthma in childhood and adulthood; this was 1.6 times at age 44 years when spirometry measurements were used [21]. In addition, a German birth cohort study that followed 1314 children from birth to 7 years of age showed that early atopic dermatitis was associated with asthma at school age [8]. However, early wheeze and sensitization were an important cofactor in this as well. Early atopic dermatitis alone without any cofactor gave no increased risk for asthma. Bergmann et al. found a strong association between atopic dermatitis in early infancy and allergic airway disease at 5 years of age in which they combined asthma and allergic rhinoconjunctivitis in their analysis [22]. In this study, a positive family history was reported as an important risk factor as well. A systemic review and meta-analysis including 17 studies found among the significant risk factors associated with the development of asthma both atopic dermatitis (OR 2.02, *p* < 0.001) and a positive family history (OR 2.20, *p* > 0.001) [23]. For peanut allergy, a strong genetic component is suggested by several research reports. For example, if a parent of sibling has peanut allergy, there is a sevenfold increase of developing peanut allergy and with monozygotic twins, there is a likelihood of 64% of peanut allergy when the sibling has a peanut allergy [24, 25].

Loss-of-function variants of the filaggrin mutation (FLG) results in an impaired epidermal barrier function and have been shown to be a risk factor for the development of atopic dermatitis, allergies, and asthma [26]. A population birth cohort study in the UK with 1.456 newborns followed children until the age of 18 years and found an increased risk of eczema and asthma (RR 2.41, *p* < 0.001) in patients with a filaggrin mutation. In patients with allergic sensitization and a filaggrin mutation, this risk was even more pronounced (RR 13.67, *p* < 0.01) [27].

## Prevention of asthma and food allergy

Besides early introduction of food allergens and optimal treatment of the skin barrier, there is not much evidence for preventive interventions for the development of food allergy and asthma. A diet rich in omega-3 fatty acids and house-dust mite avoidance were suggested, but in studies, there turned out to be no effect [28]. A Cochrane analyses reported little evidence for dietary intake or fish oil in order to improve asthma control [29]. In addition, a study with maternal supplementation of fish oil during pregnancy did not show an effect on the progression of IgE-mediated allergic disease from 1 to 6 years of age [30]. Furthermore, very low evidence was found in a recent systematic review that studied the effect of vitamin D supplementation in pregnant women, breastfeeding women, and infants on developing atopic diseases [31].

Breastfeeding remains the best advice for young babies according to the World Health Organization guidelines, but it does not appear to have a protective effect on the development of food allergy and asthma [32]. However, delaying introduction of food by exclusively breastfeeding can result in a higher risk for a food allergy [33, 34••].

Altering the microbiome has been shown to have an effect on allergic diseases like food allergy; however, results are contradictive in different studies and studies are difficult to compare because of unequal methods [21]. Therefore, more studies are needed to analyze interventions of the microbiome on the prevention of food allergy and asthma.

## Asthma and food allergy: mutual influences

As food allergy and asthma may often coincide, the question arises whether the presence of asthma may be a risk factor for the development of severe allergic reactions to foods. It is conceivable that asthma may have an unfavorable effect on accidental food allergic reactions in daily life but also on the course of an oral food challenge as part of the diagnostic approach to food allergic patients. It has been shown that food allergy is a risk factor for developing asthma. Atopic dermatitis appears to be the common denominator in this interaction, all reviewed above. There are also some studies looking at the opposite direction, i.e., asthma as a predictor for severe reactions to food. Calvani et al. demonstrated in a series of 163 children that a clinical history of asthma (OR 7.1; 95% CI 2.5–20.2) and chronic/relapsing gastrointestinal symptoms (OR 3.6; 95% CI 1.3–9.9) were associated with a greater risk of severe anaphylaxis. Asthma increased the risk for wheezing (OR 2.2; 95% CI 1.1–4.5) and respiratory arrest in allergic reactions (OR 6.9; 95% CI 1.9–32.0) [35]. In a survey of 1094, patients allergic to peanut or tree nut, life-threatening bronchospasm was most likely in patients with severe asthma (relative risk, 6.8 [4.1–11.3]) and less so in patients with milder asthma (2.7 [1.7–4.0]) [36]. In addition, in a series of 13 cases of fatal or near-fatal anaphylaxis to peanut, all patients had a history of asthma [37]. On the other hand, patients with asthma appear not to have an increased risk of severe reactions during oral food challenges [38, 39••]. The reason for this might be that oral food challenges are carried out under controlled conditions, where asthmatic patients need to be well controlled before undergoing a food challenge.

Isolated asthmatic reactions are an uncommon expression or food allergy. However, asthmatic reactions can be part of the allergic response [35, 40, 41]. During food allergic reactions, non-specific bronchial hyperreactivity (BHR) may increase. James et al. performed methacholine inhalation challenges before and after a double-blind placebo-controlled food challenge (DBPCFC). In seven of 12 patients, experiencing chest symptoms during a positive oral challenge BHR increased [42]. This phenomenon was confirmed in a recent study. In that study, it was shown that although food allergens are a rare trigger of food-induced asthmatic reactions in schoolchildren with asthma, they could enhance BHR, despite a lack of evident clinical respiratory signs and decreased in FEV1 values after food challenge [43•].

IgE directed to cross-reactive allergens may be responsible for both respiratory reactions and allergic reactions to food. Cross-reactivity between pollen and allergens from fruits and vegetables is the best-known example of the link between inhalant and food allergy. Already in 1942, this connection, later coined as oral allergy syndrome (OAS) [44], has been reported in four patients with hay fever who experienced itching of the mouth after eating raw fruits [45]. Allergic rhinoconjunctivitis based on a pollen allergy is linked with oral-pharyngeal symptoms such as oral itching and throat tightness, sometimes accompanied by gastro-intestinal symptoms. A very large series of fruits and vegetables and related pollen as birch, mugwort, and ragweed have been implicated in OAS [46]. Asthmatic reactions are not part of this syndrome [47]. Cross-reactivity between food and inhalant allergens can be associated with asthma. Van Ree at al. studied a group of 28 patients from Italy who had asthma after consumption of snail. All patients had asthma and/or allergic rhinitis caused by house-dust mite. RAST inhibition showed cross-reactivity between snail and house-dust mites [48]. In this study, tropomyosin, responsible for cross-reactivity between house-dust mites and shrimps, played a minor role. Whereas, severe asthma is the major symptom of snail allergy, shrimp allergy mainly results in skin reactions or anaphylaxis [49]. House-dust mite-induced asthma and the simultaneous presence of IgE to the house-dust mite allergens Der p 1, 2, and 10 increase the risk of shrimp allergy. Der p 10 is the mite tropomyosin cross-reacting with the major shrimp tropomyosin Pen a 1. In shrimp, allergic patient levels of IgE to Der p 10 and Pen a 1 are elevated compared with non-allergic patients [49].

## Airborne food allergens

Although allergic reactions to food commonly occur after ingestion of food, reactions to airborne food allergens are also possible. Roberts et al. described a group of 12 children with an IgE-mediated food allergy who developed asthma on inhalational exposure to food, while the offending food was being cooked. Nine children underwent a food challenge with aerosolized food solutions. Five out of nine developed objective asthma symptoms [50]. In a survey on self-reported symptoms among passengers of commercial airplanes, 41 out of 471 with a known peanut, tree nut, or seed allergy reported symptoms on an airplane. Most reactions occurred after inhalation of the food allergen, predominantly peanuts. Twenty-two percent of subjects reported hoarseness or wheezing [51].

Most asthmatic reactions to inhaled food allergens are described in occupational settings. A large systematic review identified 372 different potential causative allergens and 184 irritants or workplaces that may lead to occupational asthma [52••]. Among these were food components such as flour and alfa-amylase from bakery sites, egg proteins, Atlantic salmon, fishmeal, Norway lobster, prawn, snow crab, seafood, trout, and turbot. In addition, respiratory reactions to inhaled allergens from soybean, bell pepper, and tea dust have been described.

Wheat is one of the major food allergens that may provoke respiratory symptoms in workers exposed to flour. In one small study, adults with a history of adverse reactions to the ingestion of wheat responded to oral food challenges and a specific bronchial challenge with wheat proteins [53]. All subjects reacted to the bronchial challenge test irrespective whether they had respiratory symptoms in their history or during the food challenge. Two subjects experiencing asthma induced by ingestion of food contaminated with raw wheat, only reacted to raw wheat in the bronchial challenge test. As not all patients were working in the bakery industry, the presence of asthma induced by inhaled flour is not strictly related to occupational exposure. Asthma symptoms to inhaled wheat may also occur in subjects not displaying respiratory symptoms after wheat ingestion.

Occupational rhinitis and asthma have been demonstrated in workers exposed to lupin. Although very rare, food allergy has been reported in some of these employees [54]. Occupational respiratory allergy and concomitant food allergy have also been shown in workers exposed to inhaled egg allergens. These subjects developed rhinitis and asthma first and subsequently other allergic symptoms to ingested egg [55]. Asthma and food allergy have been seen in workers handling asparagus [56] and aniseed [57]. However, in a large study among greenhouse workers sensitized to bell pepper pollen [58], employees did not report food allergy (personal communication). Also, asthma among salt water fish processing workers was not accompanied by food allergy [59]. It is possible that the allergen itself or other factors determine the nature of symptoms. In case of bell pepper allergy, pollen is more allergenic than other parts of the plant, which may explain why consumption of bell peppers by greenhouse workers with a bell pepper pollen allergy can be tolerated.

## Conclusion

Allergic rhinitis, bronchial asthma, and atopic dermatitis are part of the atopic syndrome. IgE-mediated food allergy is an integrated component of this syndrome. Thus, food allergy and asthma often coexist. However, food allergy and asthma are interconnected with each other beyond the presence of simple comorbidity. Food allergy precedes and predisposes to asthma, and mutual interactions range from respiratory symptoms and bronchial hyperreactivity during food-induced anaphylaxis to severe asthma due to cross-reactive food allergens and to occupational asthma upon exposure to airborne allergens. The presence of asthma in food allergic patients should not be underestimated, as the combination of these two conditions may lead to severe and sometimes fatal allergic reactions.
